# Quantitative Dual-Isotope Planar Imaging of Thorium-227 and Radium-223 Using Defined Energy Windows

**DOI:** 10.1089/cbr.2019.3554

**Published:** 2020-08-31

**Authors:** Iain Murray, Bruno Rojas, Jonathan Gear, Ruby Callister, Adriaan Cleton, Glenn D. Flux

**Affiliations:** ^1^ Physics Department, Royal Marsden NHS Hospital, Sutton, United Kingdom.; ^2^ Bayer AG, Berlin, Germany.

**Keywords:** Thorium-227, Radium-223, quantitative imaging, alpha emitter imaging, theragnostic

## Abstract

***Introduction:*** Thorium-227 is an alpha-emitting radioisotope with potential therapeutic applications in targeted alpha therapy. Thorium-227 decays to Radium-223, which may have an independent biodistribution to that of the parent Thorium-227 radiopharmaceutical. Quantitative *in vivo* imaging with sodium iodide (NaI) detectors is challenging due to cross-talk between neighboring γ-photopeaks as well as scattered γ-photons. The aim of this work was to validate the use of a spectral analysis technique to estimate the activity of each isotope within a region of interest applied to a pair of conjugate view planar acquisitions, acquired at multiple energy windows.

***Methods:*** Energy spectra per unit activity arising from unscattered Thorium-227 photons and Radium-223 photons as well as from scattered photons were modeled. These spectra were scaled until the combination of these component spectra resulted in the closest match to the measured data in four energy windows.

***Results:*** Measured estimates of activity followed the known decay curves in phantoms representative of a human torso. The mean errors in estimating Thorium-227 and Radium-223 were 5.1% (range −8.0% to 40.0%) and 3.4% (range −50.0% to 48.7%), respectively. The differences between the integrals of the theoretical and estimated time activity curve were <10% for both Thorium-227 and Radium-223.

***Conclusion:*** γ-camera quantification of Thorium-227 and Radium-223 can be achieved by using multiple energy window acquisitions.

## Introduction

Thorium-227 (^227^Th) is an α-emitting radionuclide with a half-life of 18.7 days. ^227^Th decays to Radium-223 (^223^Ra), which decays by a series of further α- and β^−^-emissions to the stable isotope Lead-207, with a half-life of 11.4 days (Table 1).^1^ ^223^Ra is a calcium analogue that demonstrates increased uptake in regions of high osteoblastic activity. This has led to routine clinical application in the treatment of metastatic castrate-resistant prostate cancer patients with bone metastases and no visceral metastases.^2^ ^223^Ra also emits a number of γ-photons and several studies have demonstrated that γ-camera imaging may be used to quantitatively assess the biodistribution of ^223^Ra *in vivo*, both to normal organs and to target lesions.^3–6^

**Table 1.** ^227^Th Decay Scheme: Photon Energies with an Abundance >1% Are Shown

Decay	Decay mode	Half-life	Principal photon energies (keV) (% abundance)
^227^Th→^223^Ra	α	18.7 days	12.3 (21.0), 50.1 (8.0), 79.7 (1.7), 85.4 (1.2), 88.5 (2.0), 94.0 (1.4), 210.7 (1.1), 236.0 (12.3), 256.3 (7.0), 286.1 (1.5), 300.0 (2.3), 304.5 (1.2) 329.9 (2.7), 334 (1.1)
^223^Ra→^219^Rn	α	11.4 days	11.7 (25.0), 81.1 (15.0), 83.8 (24.9), 94.9 (11.3), 122.3 (1.2), 144.2 (3.2), 154.2 (5.6), 269.5 (13.7), 323.9 (3.9), 338.3 (2.8), 445.0 (1.3)
^219^Rn→^215^Po	α	4.0 s	11.1 (1.1), 271.2 (10.8), 401.8 (6.4)
^215^Po→^211^Pb	α	1.8 ms	
^211^Pb→^211^Bi	β^−^	36.1 min	404.9 (3.8), 427.1 (1.8), 832.0 (3.5)
^211^Bi→^207^Tl	α	2.1 min	10.3 (1.1), 72.9 (1.3), 351.1 (12.9)
^207^Tl→^207^Pb	β^−^	4.8 min	

Although ^223^Ra can be used to treat bone metastases, ^223^Ra ions do not specifically target cancer cells and therefore cannot be used to treat soft tissue metastases. For radionuclides to be taken up by specific cells, the radionuclide must be labeled to a targeting agent such as an antibody or antibody fragment that has a specific affinity to a biological target presented by the cell.

^227^Th has been successfully labeled to a number of such antibodies.^7–14^ However, ^223^Ra does not easily form stable complexes.^15^ Therefore, it is possible that after the decay of ^227^Th to ^223^Ra, ^223^Ra may dissociate from the antibody complex, resulting in free ^223^Ra ions with a biodistribution independent of the ^227^Th-labeled antibody. As these ^227^Th compounds enter clinical trials, there is a need to assess these potentially independent biodistributions.

Dual-isotope imaging can be performed with γ-cameras by using separate energy windows associated with the photopeaks of the different isotopes. In the case of these trials, a multicenter imaging protocol using the energy windows detailed in Table 2 has been implemented. The rationale for these windows is discussed by Larsson et al.^16^ However, downscatter and cross-talk between neighboring photopeaks, due to the finite energy resolution of sodium iodide (NaI) detectors, mean that quantitation of ^227^Th and ^223^Ra requires careful analysis of the photons collected in these windows.

**Table 2.** Emissions Anticipated Within Energy Windows

	75–100 keV	135–165 keV	215–260 keV	260–285 keV
^227^Th primary photons	✓		✓	
^223^Ra primary photons	✓	✓		✓
^227^Th scattered photons	✓	✓	✓	
^223^Ra scattered photons	✓	✓	✓	✓

The energy spectrum of measured counts in response to multiple radionuclides can be considered to be the weighted sum of a number of basis functions.^17^ Each function is representative of the energy spectrum associated with a subclass of photons, for example, primary photons from an isotope of particular interest.

The aim of this article is to describe and validate a spectral analysis methodology to process the data from multiple energy windows detecting ^223^Ra and ^227^Th emissions to determine the activity of each isotope within a defined region of interest (ROI).

## Methods

### Spectral analysis methodology

Gamma photons are emitted at discrete energies with probabilities of emission per unit decay, *n*(*E*), characteristic of a particular radioisotope. The measured count-rate in the presence of unknown activities of ^227^Th and ^223^Ra (*A*_Th227_ and *A*_Ra223_) can be represented as a continuous function of energy, *C*(*E*) obtained from a weighted summation of component energy spectra. The component energy spectra represent count-rates per unit activity arising from unattenuated primary photons emitted by either ^223^Ra and its daughter isotopes, *P*_Ra223_(*E*), or by ^227^Th, *P*_Th227_(*E*), as well as photons originally arising from either isotope that are scattered within the patient or the γ-camera collimator before detection, *S*_Ra223_(*E*) and *S*_Th227_(*E*) respectively.

Equation 1


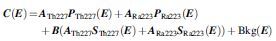


It is expected that the number of scattered photons is proportional to the activity of the isotopes. In Equation 1, the term *B* is a constant of proportionality describing this relationship. Bkg (*E*) represents a contribution to the count-rate per unit activity from background radiation. For the purposes of this investigation, it was assumed that this could be determined experimentally. If the functions *P*_Th227_, *P*_Ra223_, *S*_Th227_, and *S*_Ra223_ are known, then it can be seen that the relative weight of these component functions can be adjusted until a best fit to the background-corrected measured total counts is obtained. This section describes the derivation of these functions accounting for patient-specific attenuation, modeling of the thallium doped sodium iodide (NaI(Tl)) energy resolution, and the absolute sensitivity of the γ-camera.

**FIG. 1.**
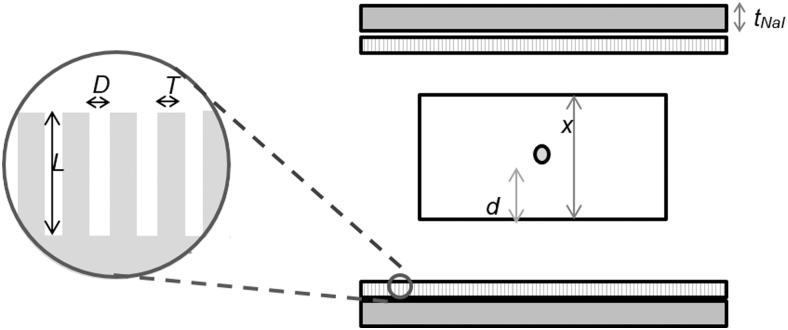
Schematic of source in γ-camera field of view.

A source of radiation within an attenuating medium (assumed to be water) of thickness *x* and attenuation coefficient *μ* was considered within the field of view of a double-headed γ-camera. The γ-camera heads comprised NaI(Tl) crystals with a thickness *t*, as well as collimators characterized by parallel holes of diameter *D*, length *L*, separated by lead septa of thickness *T* (Fig. 1).

The conjugate view technique is commonly used to quantify activity from planar images. Background-corrected count-rates are measured both anterior (*C*_ant_*(E)*) and posterior (*C*_post_*(E)*) to the patient. The background corrected geometric mean count-rate, $C_{GM}\left(E\right)$, is defined as the square root of the product of these measurements.

Equation 2


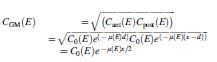


where *μ(E)* is the energy-specific attenuation coefficient of water and $C_{0}\left(E\right)$ is the theoretical count-rate in the absence of attenuation.

The relationship between *C_0_(E)* and the source activity, *A*, is determined by the probability of photon emission, *n(E)* and the sensitivity, *S*_GC_, of the γ-camera.

Equation 3


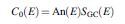


Sensitivity is determined by the proportion of photons entering the detector undergoing photoelectric absorption $\eta_{ɛ}\left(E\right)$ as well as by the geometric efficiency of the collimator, $\eta_{G}\left(E\right)$:

Equation 4


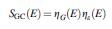


Equation 5


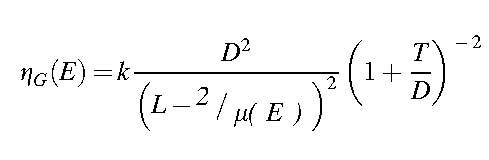


Equation 6


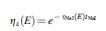


where *k* is a parameter dependent on the shape of the collimator holes, *t*_NaI_ is the thickness of the γ-camera crystal, and $\tau_{NaI}\left(E\right)$ is the energy-dependent photoelectric absorption coefficient of NaI(Tl).^18^

A model for the geometric mean count-rate per unit activity arising from a radionuclide emitting gamma photons, characterized by the spectrum, *n(E)*, was generated by substituting Equations 3–6 into Equation 2 and dividing by *A*.

Equation 7


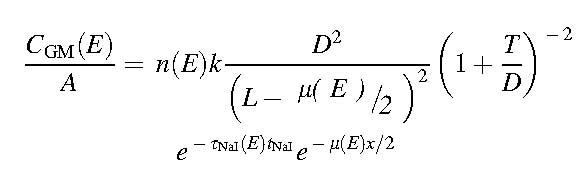


The energy resolution of the detector ER(*E*) was then modeled by convolving the result of Equation 7 with a Gaussian kernel to represent the measured geometric mean count-rate per unit activity C_GM_measured_(*E*). The width of the kernel was energy dependent, according to

Equation 8


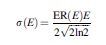


where

Equation 9


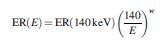


and *w* was determined empirically by analyzing measurements of energy resolution over multiple energies up to 511 keV. These measurements were made during acceptance testing and were performed according to the NEMA-1 2012 standard.^19^ For the Siemens Intevo γ-camera, *w* was found to be 0.09. Therefore,


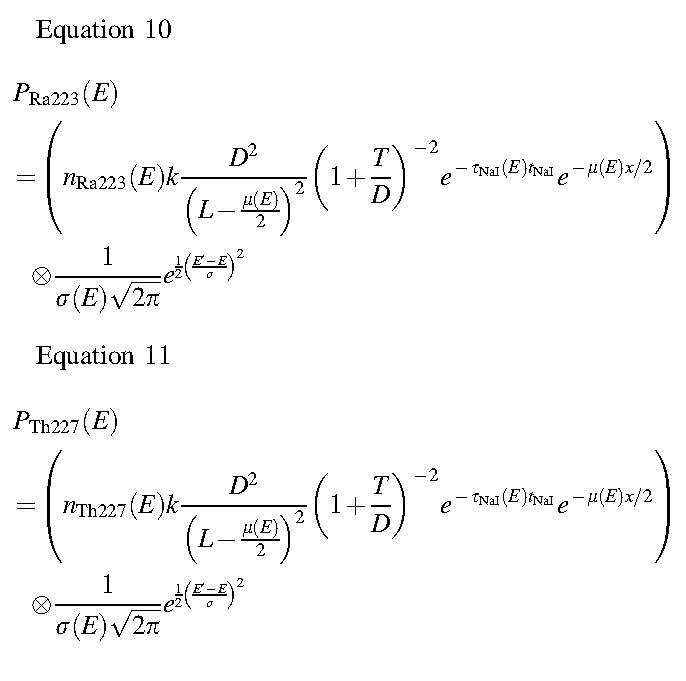


**FIG. 2.**
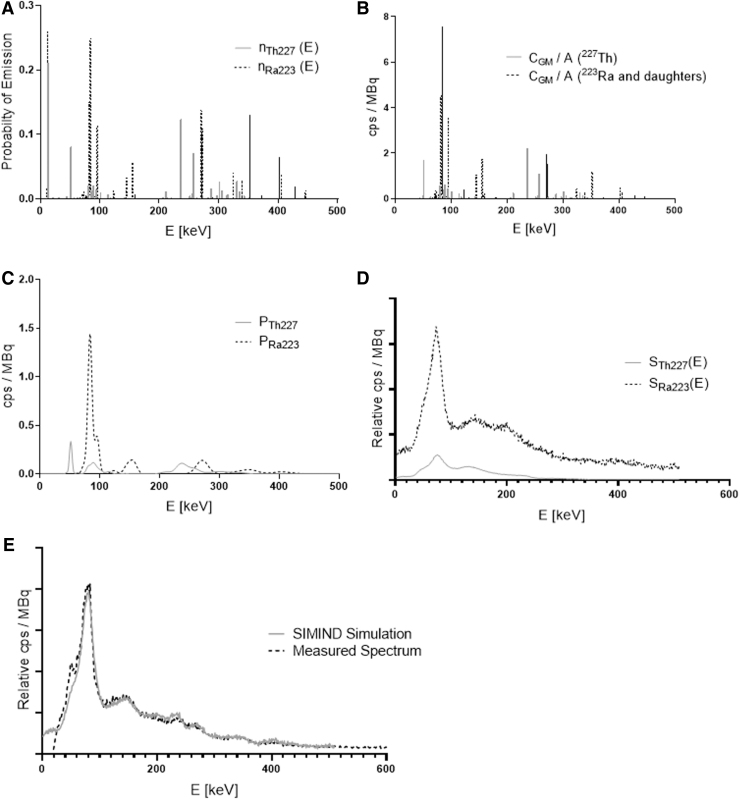
Modeling of primary photon and scattered photon energy spectrum components. **(A)** Energy spectra of emitted photons from ^227^Th as well as from ^223^Ra and daughter isotopes. **(B)** Modeled geometric count-rate *C_GM_(E)* per unit activity without consideration of energy resolution. **(C)** Modeled geometric count-rates *P_Th227_(E)* and *P_Ra223_(E)* after convolution of *C_GM_/A* with energy-dependent kernel modeling energy resolution. **(D)** Functions showing shape of scatter modeled within γ-camera field of view. **(E)** A comparison of the γ-camera spectrum observed on day 9 of imaging the Jaszczak phantom with the SIMIND simulation of a cylindrical phantom.

The analytical modeling of unattenuated photons is summarized in Figure 2A–C.

To derive the energy spectra components associated with scattered photons, initial estimates of the basic functions *S*_Th227_*(E)*, and *S*_Ra223_*(E)* were generated from energy spectra derived from Monte Carlo simulations (Fig. 2D). Simulations were performed by using the SIMIND Monte Carlo software^20^ of a water-filled cylindrical phantom (20 cm diameter and 20 cm height) homogenously filled with either ^227^Th or ^223^Ra and placed 12 cm from a Siemens Symbia γ-camera. Simulation details and input data for the simulated collimator, crystal, backscatter layer, intrinsic energy, and intrinsic spatial resolution were based on validated criteria from an earlier study^21^ and are summarized in Table 3.

**Table 3.** SIMIND Input Parameters for Simulation of a Siemens Symbia γ-Camera Detector

SIMIND input	Value
Crystal thickness (NaI(Tl))	9.5 (mm)
Backscatter thickness (PMT)	11.0 (cm)
Cover thickness (Al)	0.1 (mm)
Gap between collimator and detector	1.0 (mm)
Energy resolution (@140 keV)	9.0 (%)
Intrinsic spatial resolution (@140 keV)	3.6 (mm)
Max. scatter order	10
Cut off energy	1.0 keV

NaI(Tl), thallium doped sodium iodide; PMT, photomultiplier tubes.

Events from primary, scattered, and septal penetrating photons incident on the detector were binned according to the interaction type. An energy spectrum of all detected events less any event due to an attenuated primary photon was recorded and used to define *S*_Th227_(*E*) and *S*_Ra223_(*E*).

Thus, the background-corrected geometric mean count-rate in an acquired energy window $\int_{E_{1}}^{E_{2}}C_{GM}dE$, was modeled by weighting the photopeak and scatter energy spectra as follows.

Equation 12


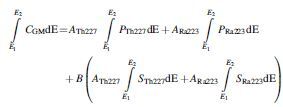


The counts due to primary photons are assumed to be directly proportional to the activity of the respective isotopes. In the case of the scattered photons, an explicit relationship between the activity of an isotope and the number of counts is not modeled. Instead, the parameter, *B*, is an arbitrary coefficient used to scale a function describing the presumed shape of the scatter spectra.

Emission spectra for all isotopes in the ^227^Th decay chain were obtained from the Brookhaven database.^1^ The NIST XCOM database was used to generate the attenuation coefficient of water as well as the attenuation due to photoelectric absorption in NaI(Tl) over the relevant energy ranges.^22^ Geometric sensitivity was defined for a high-energy collimator parameterized by hexagonal holes of 4 mm diameter and 59.7 mm length with lead septa 2 mm thick. The thickness of the NaI(Tl) detector was 9.5 mm. Values of *A*_Th227_, *A*_Ra223_, and *B* were obtained by using a generalized reduced gradient algorithm^23^ (as implemented within the solver function of MS Excel) to minimize the sum of the squared differences between the background corrected counts, and the modeled counts predicted by Equation 12 in each of the acquired energy windows.

To facilitate empirical adaption of the scatter spectrum, a modified scatter model was defined as follows:

Equation 13


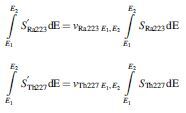


Further, it was anticipated that a “one size fits all” scatter model may not be appropriate for all scenarios. Experiments were performed to test this assumption and to determine any limits on the model used for activity estimation.

### Phantom experiments

**FIG. 3.**
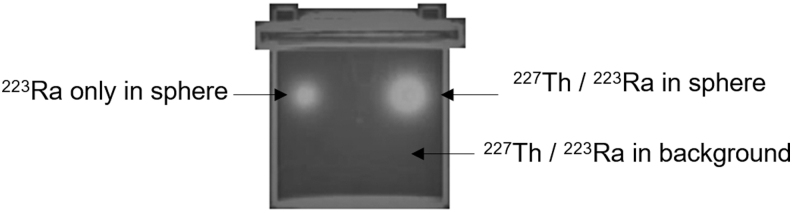
Fused planar image of Jaszczak phantom containing ^227^Th/^223^Ra anterior γ-camera image and CT scout image.

Two phantoms containing ^227^Th and ^223^Ra were used for experiments. The first was a cylindrical Jaszczak type containing two spheres of volume 14.6 and 83 mL, respectively (Fig. 3). This was selected to be representative of a patient. The smaller sphere was filled with 0.15 MBq ^223^Ra. A vial of ^227^Th was received within 12 h of purification. This solution was used to fill the 83 mL sphere with 0.54 MBq of ^227Th^, in addition to 0.014 MBq ^223^Ra. The same solution was also added to the water-filled background compartment of the phantom such that it contained 0.98 MBq ^227^Th, in addition to 0.025 MBq ^223^Ra build-up. Therefore, the sphere to background ratio was ∼40. Initial measurements of activity were made on a Capintec CRC-15R calibrator before dispensing by weight. The calibrator had previously been set up with factors traceable to the National Physics Laboratory (NPL) for both ^223^Ra and ^227^Th.^24^

The second phantom was a flat sensitivity phantom (100 × 100 × 5 mm) filled 9 days after purification with 1.33 MBq ^227^Th as well as the build-up associated with 0.92 MBq of ^223^Ra. These activities were also confirmed with an NPL traceable calibrator before filling the phantom. This phantom was used to explore the effectiveness of the activity quantification.

The Bateman equations^25^ describing the relationship between the activity of a parent and daughter radionuclide were used to calculate the activity in each phantom compartment as a function of time. Phantoms were imaged on a double-headed Siemens Intevo SPECT-CT system. The phantom was positioned in the center of the imaging couch. Anterior and posterior images were acquired for 20 min. Counts were acquired at energy windows 75–100, 135–165, 215–260, and 260–285 keV. The image matrix was 64 × 64 (pixel size of 9.59 × 9.59 mm). After planar imaging, a CT scout image of the phantom was also acquired to estimate the amount of attenuating tissue between the detector heads. The equivalent thickness of water, *x*, within an ROI was derived from the mean pixel value, *p*, of the scout image across that ROI according to Equation 14. This equation provides an estimate of the thickness in cm and was derived empirically by analyzing CT scout images of increasing thicknesses of solid water and included the effect of the patient couch.

Equation 14


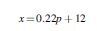


Background subtraction of the γ-camera images was performed by using counts acquired over 9 h and scaled to the phantom acquisition duration.

The Jaszczak phantom was initially imaged on the day of filling (day 0). Further images were acquired at 1, 2, 3, 4, 7, 9, 11, 14, 16, 22, 24, and 29 days after filling.

The ROIs larger than the spheres themselves were placed over the spheres within the body of the phantom to account for spill out due to the partial volume effect. Identically sized background ROIs were placed inferiorly to the spheres to subtract counts arising due to underlying and overlying activity within the background compartment. The same process was used for all energy windows, views, and imaging time points.

Values for the set of scaling coefficients ν were determined empirically by minimizing the difference between the modeled counts (using the known activities as *a priori* information) and the measured counts arising from the larger sphere. Data from day 0 were used to define the ^227^Th coefficients ν_*Th227 75–100keV*_, ν_*Th227 135–165keV*_, ν_*Th227 215–260keV*_, and ν_*Th227 260–285keV*_ at the point of minimal ^223^Ra build-up. Data from day 14 were used to subsequently define the ^223^Ra coefficients ν_*Ra223 75–100keV*_, ν_*Ra223 135–165keV*_, ν_*Ra223 215–260keV*_, and ν_*Ra223 260–285keV*_. These coefficients were subsequently applied during the analysis of data from all other imaging time points.

Quantification accuracy was assessed at each time point with respect to the known activities inside the spheres as well as with respect to the integral of the known time activity curve. The trapezoid integration method was used to measure the cumulated activity.

Additional acquisitions were also obtained on day 2 and 29 with 4 cm solid water placed between the camera and the Jaszczak phantom to test the activity estimation technique on a phantom geometry not used for calibration purposes.

Pixel-by-pixel analysis of the anterior phantom images acquired on day 22 was performed to demonstrate the potential of the technique to produce distinct images of the ^227^Th and ^223^Ra distributions.

To investigate the effect of differing geometries, the sensitivity phantom was imaged on day 9, 11, 16, 18, 22, 30, 34, and 42. The accuracy of the spectral analysis was tested by comparing the activity estimates of at each time point with the known activities in the phantom. On day 16, additional images were acquired with slabs of solid water ranging from 20 to 120 mm placed between the sensitivity phantom and the γ-camera. The known activity of the phantom was used to define scatter scaling coefficients ν_R*a223 75–100keV*_, ν_*Ra223 135–165keV*_, ν_*Ra223 215–260keV*_, and ν_*Ra223 260–285keV*_ as a function of the solid water thickness.

A further experiment was carried out to establish the effect of the distance between the sensitivity phantom and the detector. Acquisitions were performed by using a sensitivity phantom (containing 2.4 MBq ^227^Th and 1.4 MBq ^223^Ra) at distances of 2, 5, 10, 15, and 20 cm between the phantom and the detector above the couch. The posterior detector remained in a fixed position. The counts in each window were recorded. In addition, the variation in the activity estimated was analyzed as a function of source to detector distance.

## Results

**FIG. 4.**
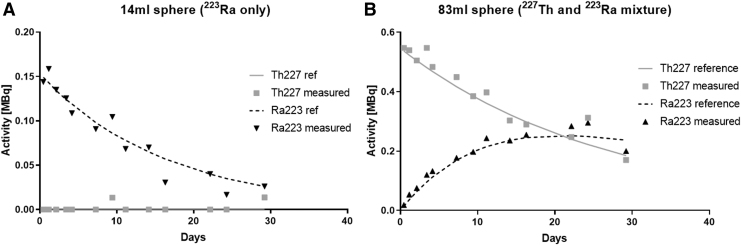
Measurements of ^227^Th and ^223^Ra within Jaszczak phantom spheres compared with ground truth activities. **(A)** 14 mL sphere containing only ^223^Ra. **(B)** 83 mL sphere containing both ^227^Th and ^223^Ra.

Application of the spectral analysis methodology resulted in estimates of activity for both ^227^Th and ^223^Ra with an average difference between the known activity and the estimated activity of 5.1% (range −8.0% to 40.0%) and 3.4% (range −50.0% to 48.7%), respectively (Fig. 4 and Table 4). In the case of the smaller sphere that was filled only with ^223^Ra, estimates of 0 MBq ^227^Th were obtained in 11 out of 13 measurements. Assessments of the integral of the time activity curves were accurate to within 10% of the ground truth values.

**Table 4.** Percentage Errors in Activity and Cumulated Activity Estimation

	14 mL sphere		83 mL sphere	
Error	^227^Th	^223^Ra	^227^Th	^223^Ra
Accuracy of activity estimate	n/a	−6.8% (range −50.0% to 10.9%)	5.1% (range −8.0% to 40.0%)	13.6% (range −15.0% to 48.7%)
Accuracy of cumulated activity estimate	n/a	−8.3%	5.3%	7.3%

The same methodology was also able to predict the activity of the phantom inserts placed in a different geometry to that used for calibration (Table 5). Despite the increased attenuation and scatter, the average difference between the known activity and the estimated activity was 8.3% (range 0.0% to 16.7%) and −11.0% (range −43.0% to 16.7%) for ^227^Th and ^223^Ra, respectively.

**Table 5.** Comparison of Measured Versus Known Activities for Case of Additional Attenuation

Time post purification (hours)	14 mL sphere				83 mL sphere			
	^227^Th activity (MBq)		^223^Ra activity (MBq)		^227^Th activity (MBq)		^223^Ra activity (MBq)	
	Expected	Measured	Expected	Measured	Expected	Measured	Expected	Measured
Day 2	0.0	0.0	0.13	0.11	0.50	0.50	0.07	0.10
Day 29	0.0	0.0	0.03	0.04	0.18	0.15	0.24	0.20

**FIG. 5.**
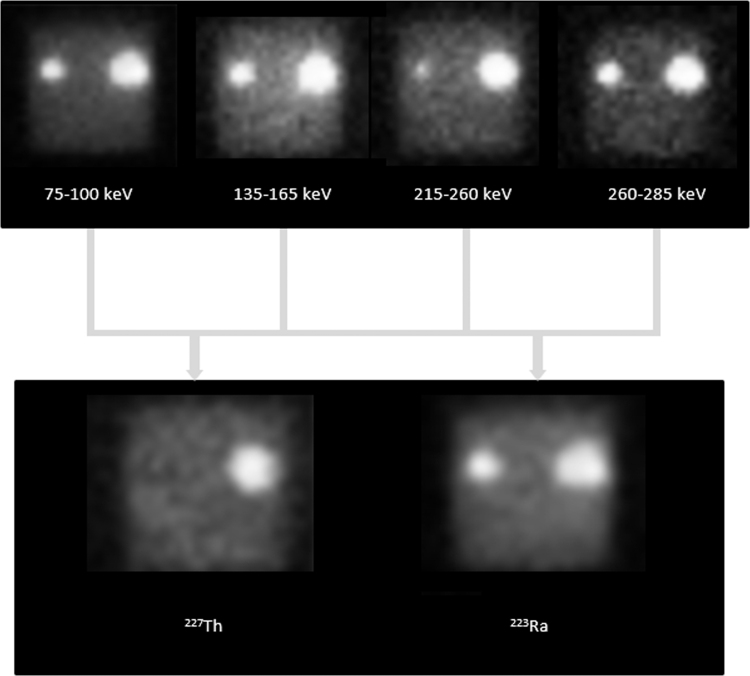
Demonstration of pixel-by-pixel analysis resulting in successful separation of ^227^Th and ^223^Ra distributions.

Pixel-by-pixel analysis of the acquired images could be used to produce images of the distribution of each respective isotope. This is demonstrated in Figure 5. The smaller sphere containing ^223^Ra only is seen in all energy windows. However, the sphere is not seen in the ^227^Th image.

**FIG. 6.**
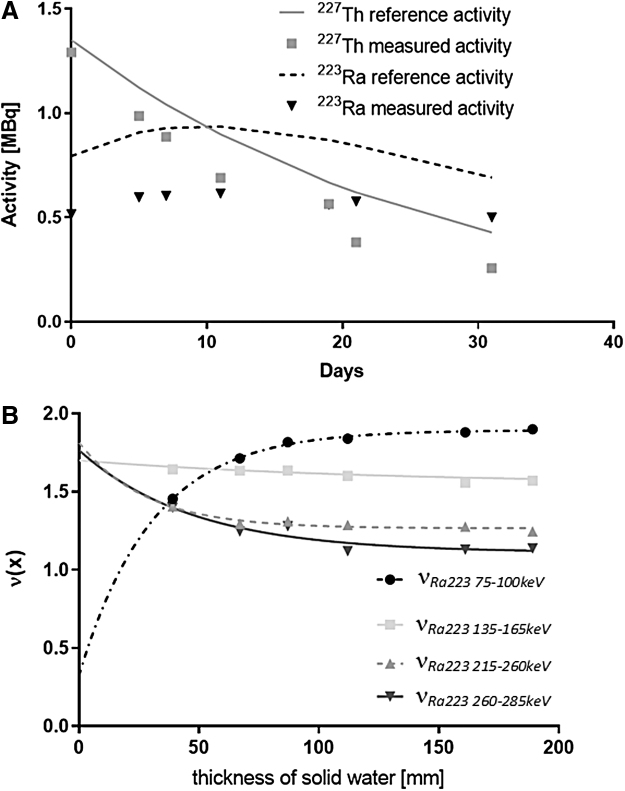
**(A)** Accuracy of activity estimation for sensitivity phantom using proposed scatter model. **(B)** Scaling coefficients ν_*Ra223 75–100keV*_, ν_*Ra223 135–165keV*_, ν_*Ra223 215–260keV*_, and ν_*Ra223 260–285keV*_ as a function of the amount of attenuating water between γ-camera heads.

Analysis of the flat sensitivity phantom data acquired over multiple timepoints demonstrated a systematic underestimation of both ^227^Th and ^223^Ra (21% and 33% respectively) with respect to the known activities in the phantom (Figure 6A).

The scaling coefficients required to correct this underestimate are demonstrated in Figure 6B as a function of the amount of scatter material between the camera heads. As the amount of scatter material increases, the coefficients become consistent and appear to stabilize.

**FIG. 7.**
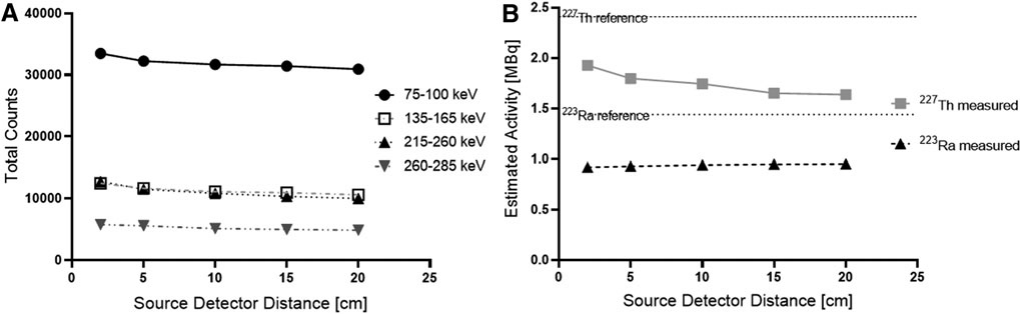
**(A)** Variation of acquired counts as a function of source to detector distance. **(B)** Estimated ^227^Th and ^223^Ra activity as a function of source to detector distance.

The effect of changing the distance between the sensitivity phantom and the detector head is demonstrated in Figure 7. At close distances there was an increase in the counts detected within all energy windows. For estimation of ^223^Ra activity, this did not result in a change in the estimated activity. For ^227^Th, a relative increase in the estimated activity of up to 18% was observed as the distance between the source and detector decreased.

## Discussion

In this work, we have attempted to develop a methodology to simultaneously quantify ^227^Th and ^223^Ra using planar γ-camera imaging. This is challenging, primarily due to the low activities administered to patients currently participating in clinical trials of ^227^Th-labeled antibodies. Planned activities typically range between 1.5 MBq – 6.0 MBq. We observed count-rates of ∼10.0 cps/MBq within the 215–260 keV window due to ^227^Th photons and ∼70 cps/MBq within the 75–100 keV window arising from ^223^Ra. Consequently, long acquisition times are required. Current ^227^Th-targeted α-therapy study design allows for up to 1 h of imaging time. Therefore, quantitation may be limited to organs within the thorax, abdomen, and pelvis, which can typically be covered by two fields of view. Low count-rates also lead to the use of larger pixel sizes, resulting in poorer spatial resolution and the increased potential for partial volume effects.

To quantify planar γ-camera images, the effect of attenuation and scatter must be accounted for. In this work, attenuation was modeled by an analytical model of photon transport within the patient and the γ-camera. Conventional scatter techniques such as the triple energy window correction are inappropriate due to the proximity of the respective photopeaks of ^227^Th and ^223^Ra.

Instead, an empirical model of the shape of the scatter spectra was scaled along with the modeled spectra of attenuated ^227^Th and ^223^Ra photons to fit the measured data to quantify planar conjugate images of ^227^Th and ^223^Ra distributions. Therefore, the accuracy with which the shape of the scatter spectra is known will affect the accuracy of the technique. The basis for our scatter spectra was a Monte Carlo simulation of a cylindrical phantom. When applying this model to a flat sensitivity phantom, systematic biases in estimated activity were observed for both ^227^Th and ^223^Ra. That is, the appropriateness of the scatter model to the patient anatomy must be considered. However, as increased amounts of scatter material were placed around the sensitivity phantom, the *ad hoc* changes to the scatter model required for accurate quantification stabilized at thicknesses above 100 mm. Indeed, when additional scatter material was placed around the Jaszczak phantom, no such biases were observed when estimating activity within the spheres. For the adult population participating in these clinical trials, patient thickness is expected to be greater than 100 mm.

There are a number of factors that lead to uncertainties in the activity estimates produced by this method. The analytical model for primary photon detection relies in the first instance on accurate knowledge of the gamma and x-ray photon emissions associated with each isotope. We used the Brookhaven database as a source of this information. More recently, Collins et al. published revised figures detailing the intensities of γ-photons emitted by ^227^Th.^26^ Comparison of these measurements against the Brookhaven database shows a discrepancy between 75–100 keV due to the absence of x-ray emissions from the Collins publication but minimal differences (<0.5%) when the convolved spectra is integrated over the remaining energy windows.

Greater uncertainty is likely to result from the fitting of the modeled data to the measured data. As described earlier, these image acquisitions are characterized by low counts, particularly within the higher energy windows. Therefore, the uncertainty in the measured counts will translate to an uncertainty in the isotope activities arising from the model fit. A limitation of this work is that the model fit was not weighted by the varying count uncertainty across the different energy windows. For the purposes of dosimetry, uncertainty in individual data measurements may be mitigated by carrying out multiple measurements over several time points. We observed differences of up to 50% between the estimated activity and the known activity but differences of <10% between the estimated and the known time integrated activities.

We also observed that changes in septal penetration as the distance between the source and the detector was varied led to variations of up to 18% in estimates of ^227^Th activity, although estimation of ^223^Ra activity remained consistent. For this reason, we would recommend the use of an HE collimator over an ME collimator to minimize septal penetration.

There remain limitations when quantifying planar γ-camera data. Our phantom setup simulated a relatively high organ to background ratio. Organs with activity concentrations closer to background activity concentration may not result in a clear signal that can be quantified. Further, organs of interest often overlap, making separation of counts challenging.^27^ Therefore, further investigations should be made into the potential of the technique when applied to SPECT imaging. Despite the low activity administered to patients receiving ^223^Ra, a number of investigations have reported that SPECT imaging of α-emitters is feasible.^28–30^

## Conclusions

Quantification of ^227^Th and ^223^Ra activity *in vivo* is possible by using multiple energy windows on a standard double-headed γ-camera.

## Author Confirmation Statement

I.M. conceived and implemented the analytical methodology presented, analyzed the data. B.R. implemented the analytical methodology and undertook experimental work. R.C. undertook experimental work. J.G. performed Monte Carlo simulations and provided advice on the analytical methodology. A.C. contributed to the article. G.D.F. contributed to the article. All co-authors reviewed and approved the article before submission.
